# Supplementary material for: Capacitive photocharging of gold nanorods
Source: Nat Commun. 2025 Dec 3;17:139. doi: 10.1038/s41467-025-67130-8 (PMC12775523; doi:10.1038/s41467-025-67130-8)
Supplement: Supplementary file 1 — Supplementary information [file 41467_2025_67130_MOESM1_ESM.pdf]

Supplementary information to "Capacitive Photocharging of Gold Nanorods"

Felix Stete,[1] Matias Bargheer,[1,2] and Wouter Koopman[1]
[1)] *Institut für Physik & Astronomie, Universität Potsdam, Karl-Liebknecht-Str. 24-25, 14476 Potsdam, Germany*
[2)] *Helmholtz Zentrum Berlin, Albert-Einstein-Str. 15, 12489 Berlin, Germany*

SUPPLEMENTARY NOTE 1: RESONANCE BACKSHIFT AFTER CENTRIFUGATION

To exclude possible processes like gold ion dissolution and re-adsorption, we centrifuged the gold nanorods for 30 min (67000$\times g$), removed the supernatant solution and redissolved the particles in water. Subsequently, we continued recording spectra in dark. As presented in Figure 1, we observe the same backshift as for the particles whose environment was not replaced. As the solution came in contact with air and the replacing water was not desoxidized, the backshift is as strong as for the measurement with added oxygen after the charging process.

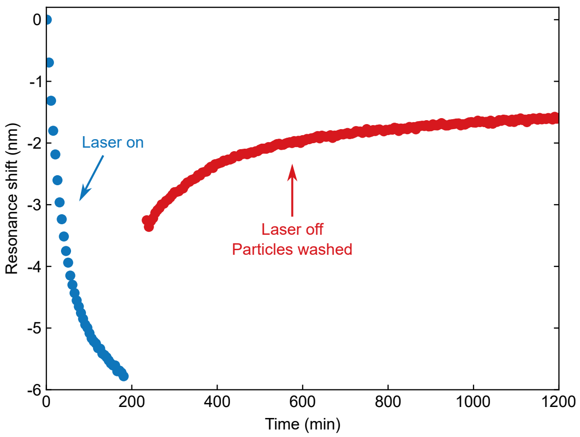

Supplementary Figure 1. **Resonance backshift after washing.** Evolution of the resonance position under illumination with a laser power of 1320 mW (blue circles) and in dark after additional particle washing by centrifugation and replacement of the supernatant. The resonance back-shifts also during the centrifugation process during which no spectra could be recorded.

SUPPLEMENTARY NOTE 2: MISFIT OF MICRO-KINETIC MODEL TO CHARGING DATA

Here, we compare our measured data with a square root behavior that was predicted previously using a microkinetic model.[1] Figure 2 depicts our data (blue circles) with the best possible square root fit ($\Delta\omega/\omega_0 = a+b\sqrt{I}$) represented by the red dash-dotted line. As a comparison the logarithmic fit from nanocapacitor model is shown as orange dashed line. The logarithmic model fits significantly better to the measured data. Possible explanations are discussed in the main text.

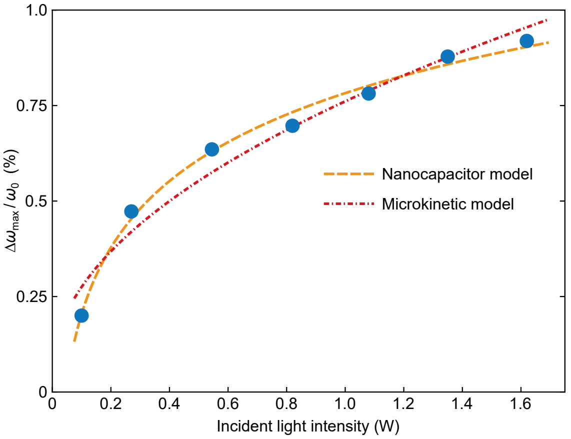

Supplementary Figure 2. **Comparison of logarithmic and square root fit.** Best fit of the microkinetic model (red dash-dotted line) and the nanocapacitor model (orange dashed line) to the measured maximum resonance shift over light intensity (blue circles).

SUPPLEMENTARY REFERENCES

[1]Kim, Y., Dumett Torres, D. & Jain, P. K. Activation Energies of Plasmonic Catalysts. *Nano Lett.* **16**, 3399–3407 (2016).
